# A dense matching method for remote sensing images fused with CPS denoising

**DOI:** 10.1038/s41598-024-59980-x

**Published:** 2024-04-23

**Authors:** Bo Zhu, Xiao Tan, Houpu Li

**Affiliations:** 1 College of Electrical Engineering, Naval University of Engineering, Wuhan, 430033 China; 2 Department of Operational Research and Programming, Naval University of Engineering, Wuhan, 430033 China

**Keywords:** Aerospace engineering, Applied physics

## Abstract

Dense matching of remote sensing images is crucial for 3D reconstruction. This study proposes an enhanced dense matching method employing the CPS image denoising algorithm, aiming to boost the SGM algorithm's accuracy and efficiency in remote sensing image matching. The stereo image pair's quality is evaluated using the PSNR index, and a decision-making criterion based on the CPS algorithm is incorporated to determine the need for denoising. Preprocessing steps, including image cropping and pixel coordinate transformation, significantly reduce computational requirements. An epipolar line model, minimizing the disparity between two pixels, is used for calculations. This model is employed to construct an epipolar image, enhancing the accuracy and efficiency of the process. The study conducted experimental validation and analysis of the mismatch rate, running time, and denoising effect of the algorithm using the Middlebury 2021 stereo datasets. Additionally, the matching results of the World-View3 satellite stereo image pairs were visualized and analyzed. The experimental results indicate that the proposed algorithm reduces the average mismatch rate by 13.1% and increases the running speed by about 3 to 4 times compared to the SGBM algorithm. Specifically, the denoising effect reduces the mismatch rate of the reconstructed image by an average of 8.97%. The results indicate that the CPS method effectively addresses dense matching challenges in the presence of image blur and noise, thereby improving the operational efficiency and accuracy of the dense matching algorithm.

## Introduction

The evolution of high-resolution stereo mapping satellites has markedly increased the use of remote sensing image as a vital data source for procuring three-dimensional information on the Earth's surface. The efficacy of these images, attributed to their high resolution and wide coverage, plays a crucial role. In photogrammetry, a pivotal challenge is remote sensing image matching, which entails identifying corresponding point coordinates on the image plane in both left and right images of a stereo pair within their overlapping area. This issue is not only significant but also widely applicable across diverse three-dimensional domains.

Current image-dense matching algorithms are broadly classified into four categories: Locally Optimal Dense Matching, Globally Optimal Dense Matching, Semi-Global Dense Matching methods (including Semi-Global Matching or SGM), and Deep Learning Dense Matching. These categories are delineated based on their optimization strategies^1^. Locally Optimal Dense Matching primarily involves pre-extraction of epipolar images. It computes disparity values between a target pixel point and its adjacent pixels, utilizing local disparity consistency as a constraint. The process includes calculating, aggregating the matching cost, and optimizing disparity. The efficiency and effectiveness of this algorithm significantly depend on the method and scale of localization in cost aggregation. To enhance local range selection and match reliability, studies have proposed dense matching algorithms based on trilateral filtering^2^ and segmentation trees^3^, incorporating image segmentation into the matching process. Globally Optimal Dense Matching, in contrast, approaches pixel matching collectively, treating it as an energy minimization modeling problem to achieve global matching outcomes. This method constructs an energy equation focused on maximizing homonymous point similarity and neighboring point compatibility. Core algorithmic strategies include the classical winner-take-all approach, confidence propagation, and dynamic programming, with improvements in confidence propagation algorithms enhancing match robustness^4^. Semi-Global Dense Matching addresses the time and memory constraints of global matching. Notable developments include the classical Semi-Global Matching (SGM) algorithm^5^, which has been refined for better matching cost computation. Techniques based on the Census transform^6,7^ and the Object Square-based Semi-Global Matching (OSGM) algorithm^8^ have shown particular effectiveness in remote sensing image matching. The OSGM algorithm and the pyramid matching strategy-based tSGM algorithm have been adapted for superior performance in this field^9^. To improve matching in weakly textured and disparity-jump areas, an SGM algorithm combined with Upright Speed Up Robust Features (U-SURF) was introduced^10^. This has spurred various SGM algorithm improvements, particularly for Digital Surface Model (DSM) production^11,12^. Advanced DSM generation algorithms employing Vertical Line Locus (VLL) with random propagation^13^ and Multi-View Vertical Line Locus (MVLL) with semi-global constraints^14^ have also been developed. Finally, the integration of deep learning in dense matching utilizes 3D convolutional neural networks to derive disparity maps from stereo image pairs^15–17^. Despite its potential, this method faces limitations in training sample time consumption and the size of training sets.

In summary, the Semi-Global Matching (SGM) algorithm currently represents the pinnacle of matching efficiency and has demonstrated superior performance in the 3D reconstruction of remote sensing images. Nonetheless, there is significant scope for enhancing the original SGM algorithm. Addressing the challenges posed by high resolution, extensive coverage, and noise interference typical in remote sensing images, this study introduces an advanced SGM algorithm. The proposed method initially applies the Cauchy proximal splitting algorithm for denoising, followed by image segmentation and pixel coordinate transformation. It then employs the epipolar line model to reduce disparities at both upper and lower ends, thereby constructing a refined epipolar image. The process culminates in semi-global dense matching to produce disparity maps. The algorithm's efficacy is validated using the Middlebury 2021 stereo datasets, showcasing substantial improvements in efficiency and matching accuracy, particularly in denoising. Further experimental validation with WorldView-3 satellite stereo image pairs confirms the algorithm's enhanced applicability for dense matching in remote sensing images.

## Fundamentals of the SGM algorithm

The SGM algorithm integrates the principle of energy function optimization, with the objective of ascertaining the optimal disparity for each pixel to minimize the global energy function of the image. This process is structured into four distinct stages: initial cost calculation, cost aggregation, disparity calculation, and disparity optimization.

### Initial cost calculation

The original SGM algorithm employs Mutual Information (MI) for cost computation, a process characterized by its complexity and specific iteration requirements, which can compromise efficiency. In response, various strategies have been developed to optimize cost calculation. A notable example is the Semi-Global Block Matching (SGBM) algorithm, integrated into OpenCV, a widely-used open-source computer vision library. The SGBM algorithm is recognized for its computational simplicity and enhanced matching efficiency. It implements the Block Truncation (BT) strategy, effectively reducing errors related to the discretization of image samples from an information sampling standpoint. Additionally, the SGBM algorithm incorporates a preprocessing stage in the cost calculation, utilizing the Sobel operator for initial image processing. The algorithm combines the BT cost of the preprocessed image with the BT cost of the original image to formulate the initial cost, which is calculated using the following equation:

1 $$ \begin{gathered} Sobel(x,y) = 2[P(x + 1,y) - P(x - 1,y)] + P(x + 1,y - 1) \hfill \\ - P(x - 1,y - 1) + P(x + 1,y + 1) - P(x - 1,y + 1) \hfill \\ \end{gathered} $$

2 $$ C(p,d) = C_{original}^{BT} (p,d) + C_{sobel}^{BT} (p,d) $$

### Cost aggregation

Pixel-by-pixel cost calculations often result in ambiguous boundaries and are prone to false matches due to noise and other factors. To mitigate these issues, an additional constraint is implemented to facilitate smoothing by imposing penalties on variations in neighboring pixel differences. This approach integrates the pixel-by-pixel cost with smoothing constraints within the energy framework, represented by E(D), for the disparity image D. This energy, E(D), encapsulates the overall energy of the image.

3 $$ E(D) = \sum\limits_{p} {(C(p,D_{p} ) + \sum\limits_{{q \in N_{p} }} {P_{1} T[\left| {\left. {D_{p} - D_{q} } \right|} \right. = 1]} + \sum\limits_{{q \in N_{p} }} {P_{2} } T[\left| {\left. {D_{p} - D_{q} } \right| > 1} \right.])} $$

In light of the aforementioned formulation, the challenge of stereo matching is redefined as identifying the disparity image D that minimizes the energy E(D). However, this task becomes non-deterministic when approached in two dimensions. The SGM algorithm circumvents this complexity by transforming the two-dimensional cost aggregation challenge into a one-dimensional problem, executed across multiple paths. This transformation involves selecting a specific path, calculating the matching cost within an effective disparity range for that path, and then aggregating the matching costs of all paths for a given pixel. The aggregate costs are summed, and the minimum value within the effective disparity range is selected as the representative matching value. The cost aggregation for any pixel p along a specific path r is formulated as presented in Eq. (4).

4 $$ L_{r} (p,d) = C(p,d) + \min \left\{ {\left. {\begin{array}{*{20}c} {L_{r} (p - r,d)} \\ {L_{r} (p - r,d - 1) + P_{1} } \\ {L_{r} (p - r,d + 1) + P_{1} } \\ {\mathop {\min }\limits_{i} L_{r} (p - r,i) + P_{2} } \\ \end{array} } \right\}} \right. - \mathop {\min }\limits_{i} L_{r} (p - r,i) $$

**Figure 1:**
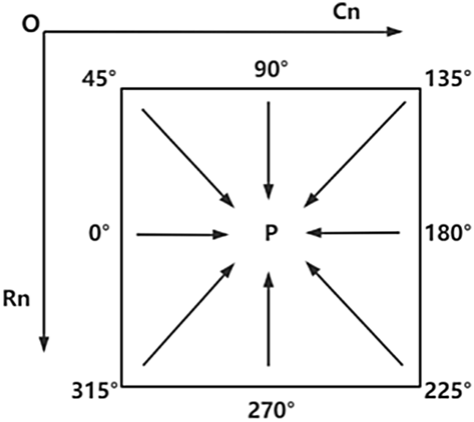
Schematic diagram of path aggregation. The arrows in the figure indicate aggregation from 8 directions.

The formula under consideration comprises three components: the first term corresponds to the matching cost, the second term is the smoothing term, and the third term is an adjustment to ensure the total matching cost does not exceed the upper limit, defined as L ≤ Cmax + P2, which represents the sum of matching costs across all paths. Opting for eight general paths has been demonstrated to enhance computational accuracy compared to four paths, while also improving computational efficiency relative to sixteen paths. Figure 1 illustrates the process of path aggregation, providing a visual representation of this procedure.

### Disparity calculation

Following the initial cost computation and subsequent cost aggregation, the disparity is determined using the Win Takes All (WTA) strategy. In this approach, the point exhibiting the lowest aggregated cost is selected as the matching point for pixel p within the disparity search range. The corresponding disparity, denoted as d, is then ascertained. Specifically, for the epipolar image, disparity d is calculated as the difference between xl and xr, where xl and xr represent the column coordinates of the left and right images, respectively.

### Disparity optimization

1. Consistency check

Consistency checking serves as a crucial technique for identifying mismatched and occluded points in image analysis. This method involves initially selecting the left image as the reference to compute its corresponding disparity map, denoted as D. Using D, the homologous image point q, corresponding to any given pixel point p in the left image, is determined. Following this, the right image is designated as the reference, and the analogous process is employed to identify the homologous image point q′, corresponding to q. According to the principle of disparity uniqueness, q and q′ are expected to align with the same pixel. Equation (5) is utilized to ascertain the consistency across all pixels.

5 $$ \left| {\left. {D_{l} (p) - D_{r} (p - (D_{l} (p),0))} \right| > \lambda_{0} = 1} \right. $$

The anomalies, encompassing both mismatch and occlusion points, are identified when the difference between two comparative metrics exceeds a threshold λ_0_. These anomalies can significantly affect the matching results. To mitigate this impact, invalidating the disparity associated with these points is recommended. The method to differentiate between these two types of anomalies is delineated in Eq. (6).

6 $$  \begin{gathered} p^{\prime} = p - ({\text{D}}_{l} (p),0) + (D_{r} (p - (D_{l} (p),0)),0) \hfill \\ \left\{ {\begin{array}{*{20}c} {D_{l} (p) < D_{l} (p^{\prime}){\text{occlusion points}}} \\ {D_{l} (p) \ge D_{l} (p^{\prime}){\text{mismatch points}}} \\ \end{array} } \right. \hfill \\ \end{gathered} $$

(2) Sub-pixel fitting

**Figure 2:**
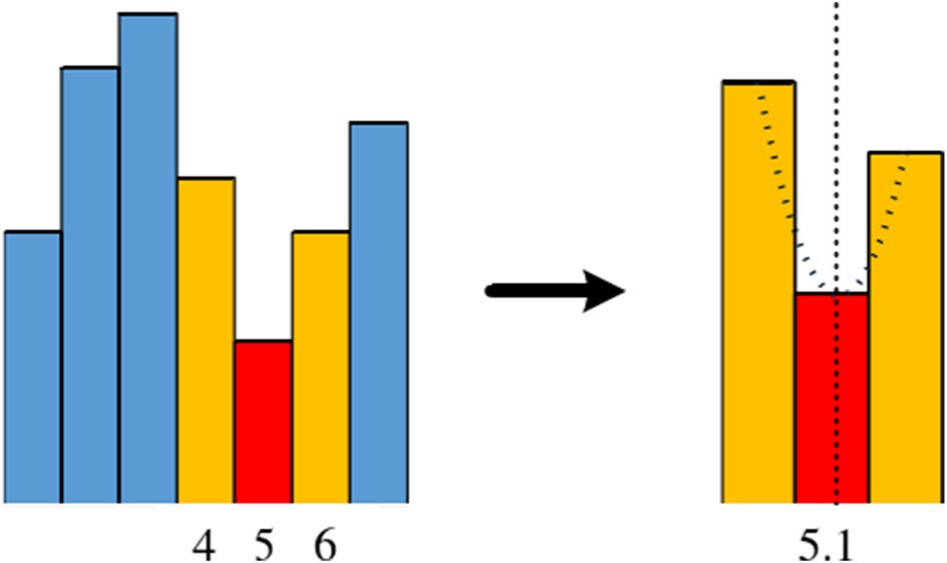
Schematic of sub-pixel fitting. The red region of the figure represents the optimal disparity value, which increased from 5 to 5.1 after sub-pixel fitting.

The disparity values derived using the Winner-Takes-All (WTA) strategy are integer-based. To enhance their precision, a sub-pixel fitting technique is applied. This process begins by identifying the optimal disparity value through the WTA strategy, followed by selecting the two adjacent disparity values. A univariate quadratic curve is then fitted based on the surrogate values corresponding to these three disparity points. The refined sub-pixel disparity values are extracted from the curve's extreme points. Figure 2 depicts the sub-pixel fitting process.

(3) Uniqueness check

In disparity calculation using the Winner-Takes-All (WTA) strategy, the occurrence of multiple minima in matched surrogate values, or minimal differences between the primary and secondary minima, can undermine the reliability of the derived disparity. Consequently, pixels failing to meet the disparity uniqueness criterion are typically excluded.

(4) Removal of small connectivity zones

Small connected regions within a disparity map are often attributable to noise. The map is formed by pixels associated with a block of a certain size. To rectify this, the connected area's size in the disparity map is calculated, and a specific threshold is set. Regions smaller than this threshold are eliminated, resulting in the assignment of an invalid disparity to the entire block.

(5) Median filter

The essence of median filtering lies in replacing the value of a pixel in the image with the median value of its neighboring points. This technique effectively brings the values of adjacent pixels closer to their true value, thus eliminating isolated noise points and enhancing the smoothness of the disparity map. The filtering process is outlined in Eq. (7).

7 $$ D(p) = \frac{1}{n}\sum\limits_{q \in N} {D(q)} $$

## Semi-global dense matching improvement methods incorporating CPS denoising

Due to the strong practical value of the SGM algorithm, many scholars have proposed improved methods to the original SGM algorithm, which mainly have optimized the initial cost calculation and cost aggregation process, i.e. the optimization of the initial cost calculation formula and the objective function of dense matching, but the difference between the optimization effect of different improvement methods is relatively small. Combined with the characteristics of remote sensing images such as high resolution, wide coverage, and noise interference, the algorithm proposed in this paper adds some image preprocessing processes based on the SGBM algorithm in the OpenCV open-source computer vision library, including image denoising, image cropping and pixel coordinate conversion, constructing epipolar images, through these preprocessing steps, the matching efficiency and accuracy of the SGM algorithm applied to remote sensing images are improved.

### Image denoising based on the Cauchy proximal splitting algorithm

The CPS algorithm demonstrates exceptional proficiency in solving convex optimization problems, exhibiting robust convergence performance. It adeptly addresses the L1 regularization problem, making it highly effective for image denoising applications. The CPS algorithm distinctively suppresses noise while preserving essential image details and texture, thereby offering considerable performance benefits^18,19^. The underlying principle of the CPS algorithm involves reformulating the image denoising challenge into a convex optimization problem using the generalized Lagrangian method. It employs proximal splitting techniques, such as Douglas-Rachford splitting, forward–backward splitting, or alternating-direction multiplier splitting, to execute iterative computations. The denoising process reaches completion upon convergence of these computations. In the context of semi-global dense matching, the CPS algorithm proves especially valuable in low-quality images with noise interference, enhancing the clarity and detail of objects, and thus improving matching accuracy. In practical scenarios, it is vital to evaluate the impact of image quality, noise, and other factors on the matching algorithm. This evaluation guides the decision to implement the CPS algorithm. In instances of high image quality, the application of this algorithm might be unnecessary. Therefore, the quality of the stereo image needs to be assessed prior to matching. As there is no reference image for remote sensing, direct measurement of image quality is not accessible. Considering that dense matching requires that the difference between images should not be too large, this study uses the right image in the stereo image pairs as the reference image. The Peak Signal-to-Noise Ratio (PSNR) index is then calculated based on the mean squared error between the left and right images of a stereo image pair. It is used to evaluate the quality of the image pair. If the PSNR is below a certain threshold, employing the CPS algorithm for denoising becomes imperative. The PSNR index equations are given in (8) and (9).

8 $$ MSE = \frac{1}{mn}\sum\limits_{i = 0}^{m - 1} {\sum\limits_{j = 0}^{n - 1} {[L(i,j) - R(i,j)]^{2} } } $$

9 $$ PSNR = 10 \cdot \log_{10} (\frac{MAX}{{MSE}}) $$

The width and height of the image have been defined as m and n. The grayscale of the left image has been defined as L(i,j), and the gray scale of the right image has been defined as R(i,j). MAX represents the maximum gray scale of the image and MSE represents the mean square error. The pseudo-code of the CPS algorithm, utilizing the Cauchy proximal operator and its main function, is presented as follows:

**Algorithm 1:**
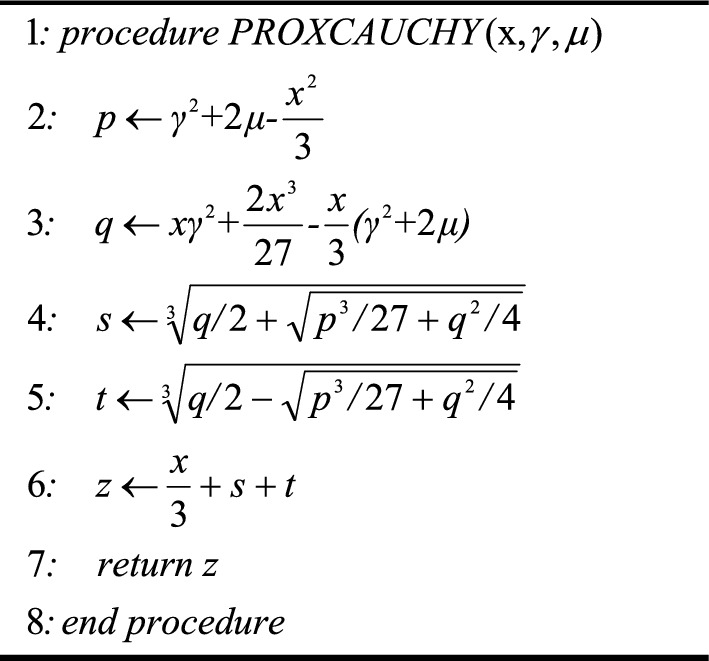
Cauchy proximal operator

**Algorithm 2:**
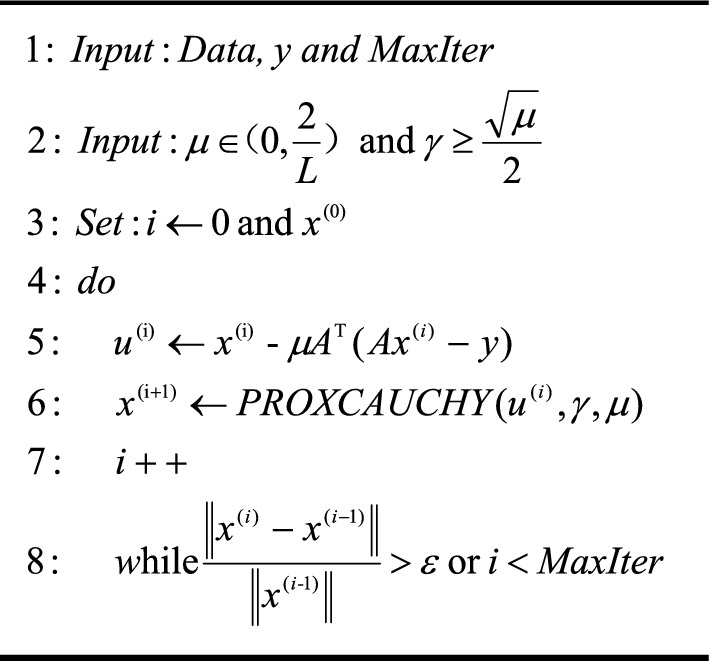
CPS Algorithm

### Image cropping and pixel coordinate conversion

When performing semi-global dense matching on stereo image pair data, it is often observed that the original remote sensing images contain redundant regions. These superfluous areas lead to decreased matching efficiency and increased computational resource utilization. The disparity map generated from semi-global dense matching solely encapsulates the disparity information between the left and right images. Addressing this disparity is intrinsically linked to minimizing the global energy function. Image cropping can markedly reduce the computational scope of this function, thereby enhancing the efficiency of solving for the disparity. Consequently, to optimize image matching efficiency, image cropping to isolate the region of interest is recommended.

**Figure 3:**
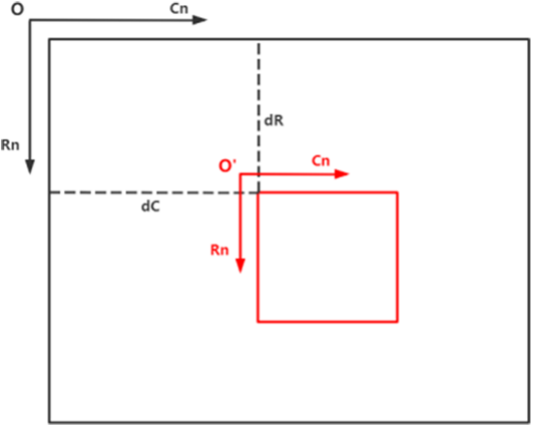
Schematic diagram of pixel coordinate conversion. The red box in the figure denotes the cropped image, while the black box depicts the original image.

After the image is cropped, the pixel coordinates of the targeted region of interest undergo a transformation. If this alteration is not adequately addressed, it can result in the generation of an erroneous point cloud in subsequent stages. Therefore, when cropping the image, it is imperative to ensure that the dimensions of the left and right images remain identical post-cropping. Additionally, it is essential to consistently use the same pixel coordinate conversion coefficients, denoted as dR and dC, across both images. This consistency is key to maintaining the accuracy of the pixel coordinates between the cropped and original images. The relationship between the pixel coordinates of the cropped image $(Rn^{\prime},Cn^{\prime})$ and the coordinates of the image before cropping $(Rn,Cn)$ can be expressed as Eq. (10), and Fig. 3 shows a schematic of the pixel coordinate conversion.

10 $$ \left\{ {\begin{array}{*{20}c} {Rn^{\prime} = Rn + dR} \\ {Cn^{\prime} = Cn + dC} \\ \end{array} } \right. $$

### Constructing epipolar images

Due to parallel projection in the motion direction and orthogonal perspective projection, linear motion transforms the epipolar line into a hyperbolic shape, while nonlinear motion generates a general curve. Consequently, correcting the epipolar line in the image is imperative before performing semi-global dense matching. Post-correction, the search process simplifies as it requires examining only the corresponding pixel points in each row. This reduction changes the search space from two-dimensional to one-dimensional, significantly boosting the matching efficiency.

In constructing the epipolar image, establishing an accurate epipolar line model is critical. The conventional approach involves segmenting the image, using affine transformation relations for matching image segments, and then utilizing the projected trajectory method to produce the epipolar image. Additionally, methods that amalgamate image-square and object-square techniques are also employed for epipolar image generation^20^. To minimize the disparity at the top and bottom of the epipolar image, this study adopts an epipolar line model that effectively reduces this disparity, thereby enhancing the accuracy and efficiency of semi-global dense matching. The principle involves directing a straight line with varying inclination angles through any pixel point on the left image and employing the forward and backward arithmetic model of the rational function to calculate the corresponding image points on the right image. The optimal inclination angle is determined when the distribution of corresponding image points on the right image for a particular angle closely approximates a straight line, allowing simultaneous determination of the left and right epipolar lines. The specific steps for constructing the epipolar image include:

- Let the inclination of the line passing through pixel p on the left image be α. From this, we can obtain the equation of the left line as $y - y_{p} = \tan \alpha (x - x_{p} )$;
- n pixel points are selected at equal intervals on the left line, and the corresponding geodesic plane coordinates are computed from the rational function inversion model of the left image $(X_{i} ,Y_{i} )$ based on their pixel coordinates $(x_{i} ,y_{i} )$, i = 1,2,…,n and different geodesic heights Z_i_;
- Calculate the geodetic coordinates of each point $(X_{i,} Y_{i} ,Z_{i} )$ from the orthonormal model of the right image and the coordinates of the homonymous image point $(x_{i} ^{\prime},y_{i} ^{\prime})$ on the right image.
- A straight line was fitted to $(x_{i} ^{\prime},y_{i} ^{\prime})$. The least squares method is used to obtain the right-hand line equation $y^{\prime} = kx^{\prime} + b$_,_ with coefficients for each:
  11 $$  \begin{gathered} k = \frac{{n\sum\limits_{1}^{n} {x_{i} ^{\prime}y_{i} ^{\prime} - \sum\limits_{1}^{{\text{n}}} {x_{i} } ^{\prime}\sum\limits_{1}^{{\text{n}}} {y_{i} ^{\prime}} } }}{{n\sum\limits_{1}^{n} {x_{i} ^{{\prime}{2}} - \sum\limits_{1}^{{\text{n}}} {x_{i} } ^{{\prime}{2}} } }} \hfill \\ b = \frac{{\sum\limits_{1}^{n} {x_{i} ^{{\prime}{2}} \sum\limits_{1}^{n} {y_{i} ^{\prime} - \sum\limits_{1}^{n} {x_{i} ^{\prime}} \sum\limits_{1}^{n} {x_{i} ^{\prime}y_{i} ^{\prime}} } } }}{{n\sum\limits_{1}^{n} {x_{i} ^{{\prime}{2}} - (\sum\limits_{1}^{n} {x_{i} ^{\prime}} )^{2} } }} \hfill \\ \end{gathered} $$

The right line inclination angle $\alpha ^{\prime} = \arctan k^{\prime}$, the root mean square of the upper and lower disparity of each pixel point is:

12 $$ D_{i} = \frac{{\left| {\left. {k^{\prime}x - y^{\prime} + b} \right|} \right.}}{{\sqrt {k^{{\prime}{2}} + 1^{2} } }}\;\sigma = \sqrt {\frac{{\sum\limits_{1}^{n} {D_{i}^{2} } }}{n}} $$

(e) Record the root mean square (RMS) of the upper and lower disparity, denoted as σ, obtained from the test. If this value represents the current minimum, document it as σ_t_ = σ and α_t_ = α;
(f) Repeat steps (1)–(5) until the evaluation is completed for each inclination angle within the predefined range for the epipolar line inclination;
(g) Utilize the parabolic equation to calculate the minimum inclination of the left epipolar line, α_min_. Subsequently, determine the right epipolar line by fitting the left epipolar line to the corresponding image point on the right image. The parabolic equation, f(α), is defined as follows:
  13 $$ f(\alpha ) = u + v \cdot \alpha + w \cdot \alpha^{2} $$

**Figure 4:**
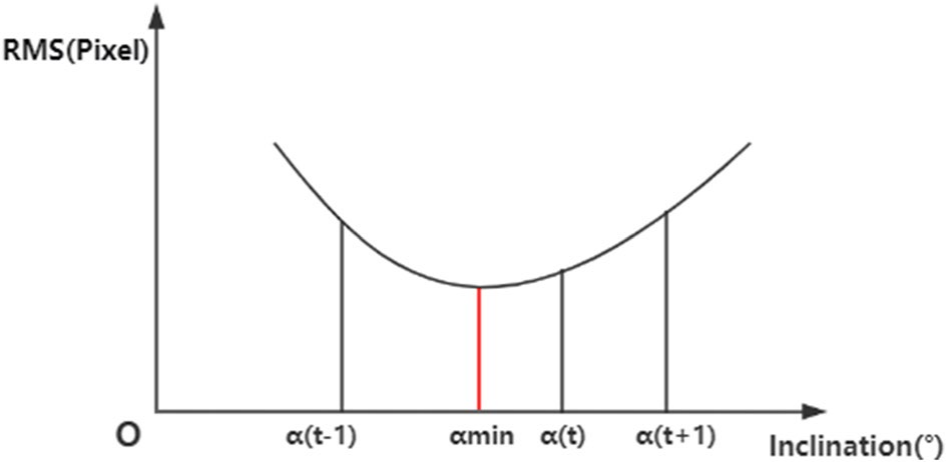
Illustration of the parabola fitting. In the figure, the abscissa represents the inclination angle, and the ordinate represents the root mean square value. The minimum value of the parabola can be determined by the coordinate values of three points.

The parameters *u*, *v*, and *w* in the equation are determined by solving a system of equations. This system incorporates α^t−1^, α^t^, α^t+1^, and their respective three sets of upper and lower disparity root mean square (RMS) values. The process of parabolic fitting is illustrated in Fig. 4.

### Algorithm flow of this paper

**Figure 5:**
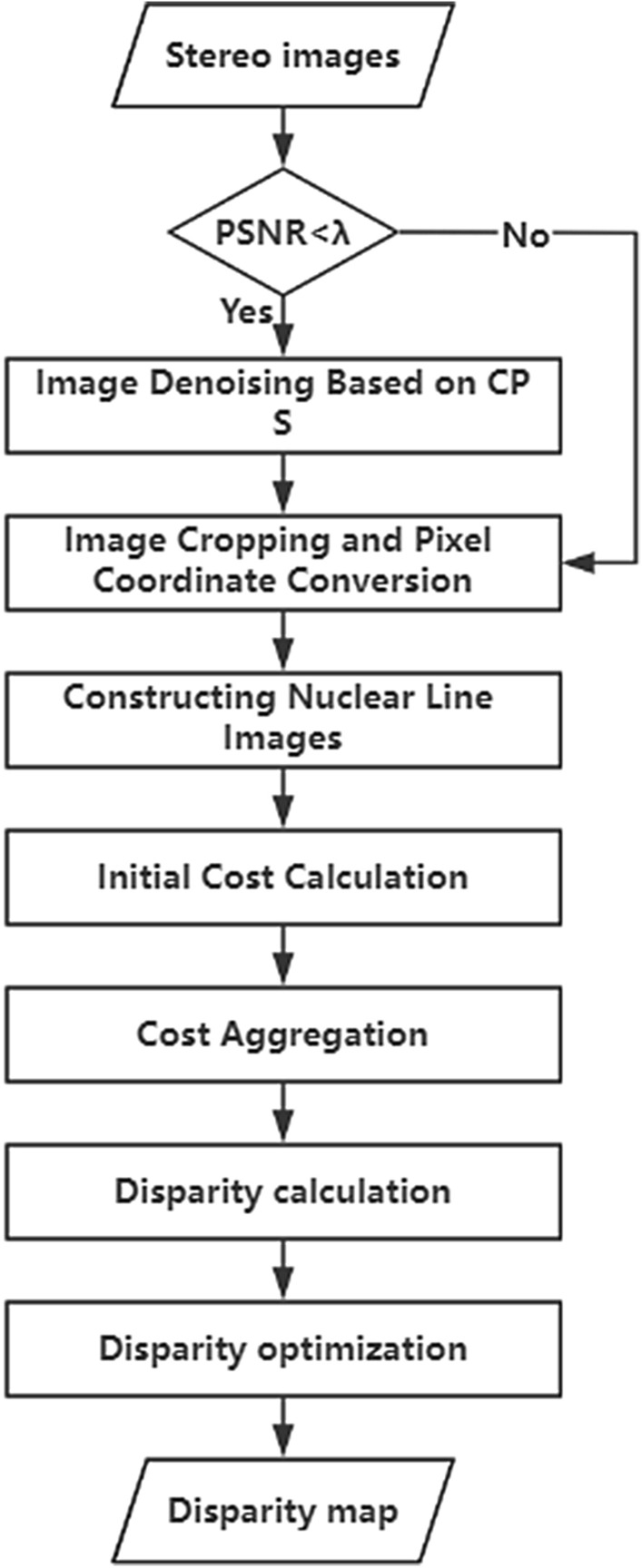
Flowchart of the algorithm in this paper.

After the improvement of the original SGM algorithm, the flow of the remote sensing image dense matching algorithm in this paper is shown in Fig. 5.

## Experimental results and analysis

### Algorithm efficiency analysis

**Figure 6:**
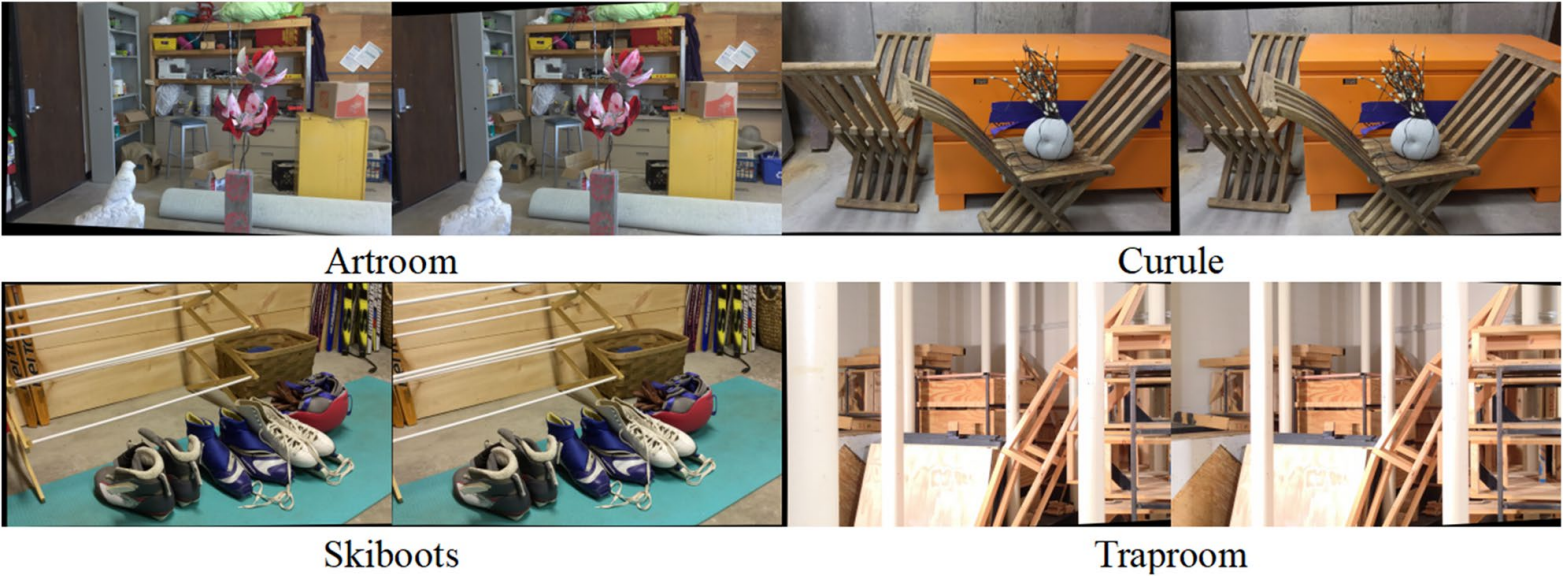
Test data from Middlebury 2021 stereo datasets.

**Figure 7:**
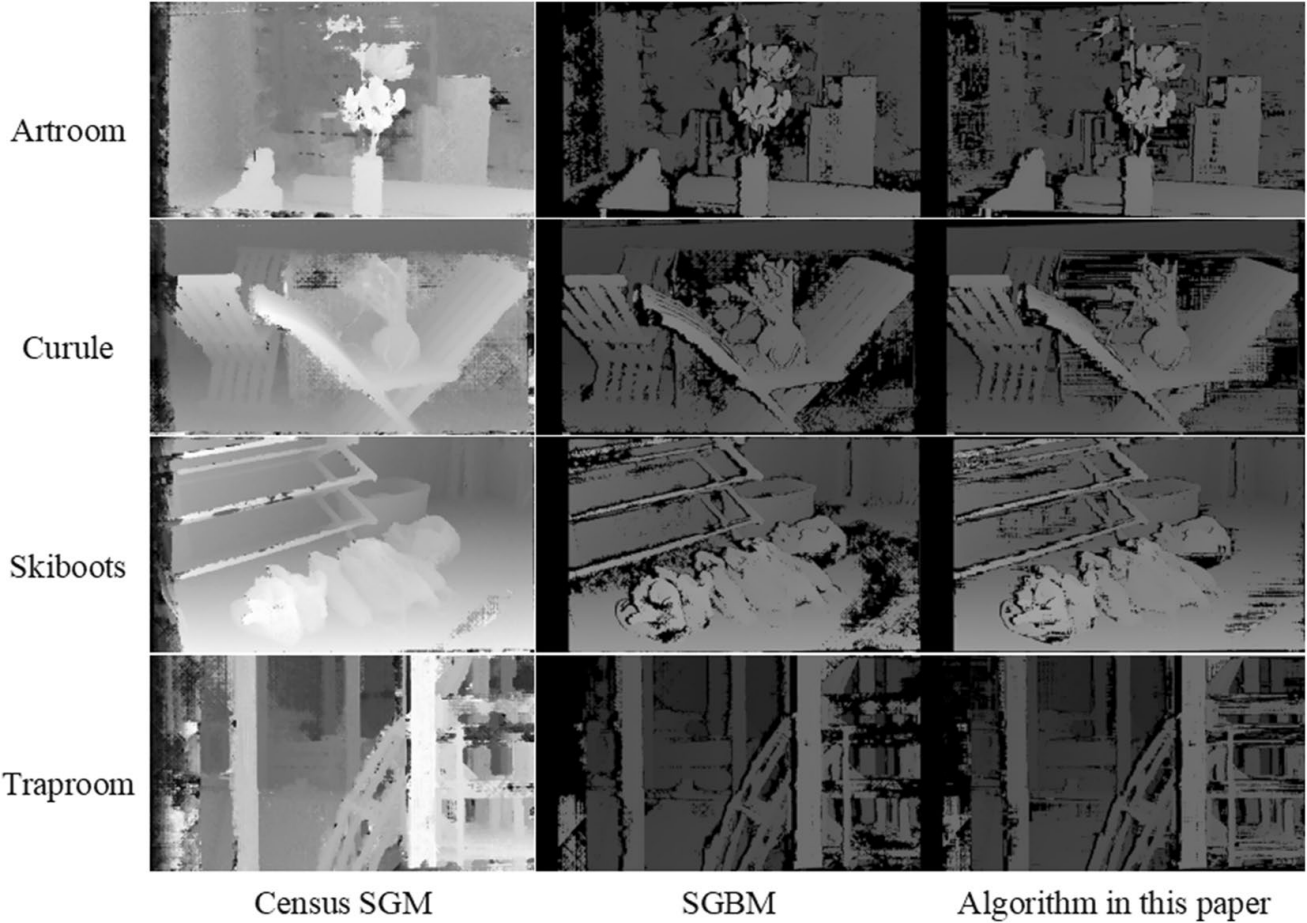
Disparity map of test data. The figure displays the results of the three algorithms applied to the Middlebury 2021 stereo datasets in the form of a disparity map.

The Census SGM algorithm, SGBM algorithm, and the algorithm proposed in this study were implemented in Python on a PC equipped with 16 GB RAM and an Intel(R) Core(TM) i5-10200H (2.40 GHz) processor. Given that ground truth, essential for calculating the mismatch rate, are unattainable from remote sensing images, this study employed the fullsize images from the Middlebury 2021 stereo datasets. These images were used to compute the algorithms' mismatch rate and execution times. In the subsequent sections, Fig. 6 displays the test data from Middlebury 2021 stereo datasets, while Fig. 7 shows the disparity maps generated by each of the three algorithms using the test data. Tables 1 and 2 provide statistics for the mismatch rate and running time of three algorithms.

**Table 1:** Mismatch rate analysis table (%).

Experimental data	Census SGM algorithm	SGBM algorithm		The algorithms in this paper
Artroom	6.43		27.27	25.09
Curule	6.13		28.09	23.94
Skiboots	6.18		26.62	21.22
Traproom	7.69		25.06	22.74

**Table 2:** Running time analysis table (seconds).

Experimental data	Census SGM algorithm	SGBM algorithm		The algorithms in this paper
Artroom	1713.84		1.624	0.481
Curule	1865.24		1.607	0.401
Skiboots	2517.74		2.089	0.564
Traproom	1395.12		1.235	0.354

The experimental findings indicate that the Census Semi-Global Matching (SGM) algorithm exhibits the lowest rate of false matches, albeit with extended processing times and reduced efficiency. In contrast, the Semi-Global Block Matching (SGBM) algorithm demonstrates a false matching rate approximately 4 times that of the Census SGM algorithm, yet offers a significant improvement in processing speed, being faster by three orders of magnitude. The algorithm introduced in this study, based on the SGBM framework, shows an enhancement in both matching accuracy and processing speed. On average, the mismatch rate is reduced by 13.1%, and the processing speed is approximately 3–4 times faster. In summary, the Census SGM algorithm is more accurate and applicable for areas with dense matching on a smaller scale. However, for large-scale dense matching, considering the constraints of processing speed, the algorithm proposed in this study demonstrates greater applicability. Consequently, this algorithm is better suited for dense matching of remote sensing images.

### Analysis of the denoising effect

**Figure 8:**
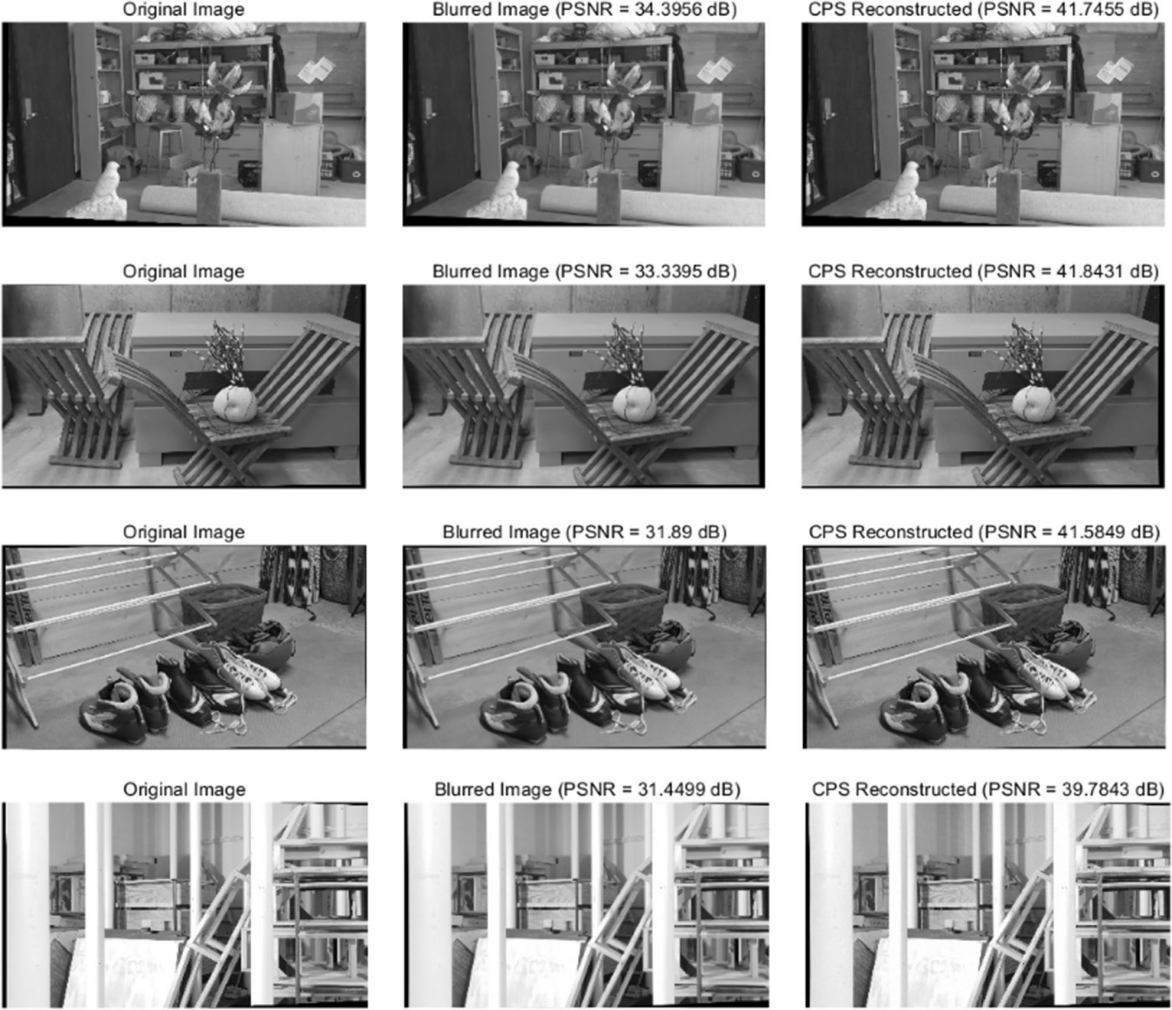
Graph of anti-noise experiment results. The central portion of the figure depicts the image after adding noise to the original image, while the right part exhibits the image after CPS denoising. Additionally, the figure presents the PSNR index used to measure the image quality.

To assess the efficacy of the algorithm proposed in this study for image denoising and anti-blurring,the test images were deliberately blurred and subjected to Gaussian noise addition. These images were then reconstructed using the CPS algorithm. The quality of the reconstructed images was evaluated using the PSNR index, where a higher PSNR index signifies superior image quality. The results of this evaluation are depicted in Fig. 8. Additionally, semi-global dense matching was applied to the reconstructed images, and the rate of false matches was compared with that of the blurred images to validate the effectiveness of the CPS algorithm. The results of this comparison are summarized in Table 3.

**Table 3:** Comparison of mismatch rate (%).

Experimental data	Blurred image	CPS reconstructed images
Artroom	29.43	27.53
Curule	31.06	29.01
Skiboots	28.35	24.31
Traproom	28.04	25.64

The experimental data indicate that the application of the Cauchy Proximal Splitting (CPS) algorithm considerably improves the quality of blurred images, evidenced by a 25.94% increase in the average PSNR index. Furthermore, the mismatch rate in images reconstructed using the CPS algorithm has decreased by an average of 8.97%. These results substantiate the effectiveness of the CPS algorithm in denoising blurred images and enhancing the matching accuracy of the semi-global dense matching algorithm.

### Visualization of matching results

**Figure 9:**
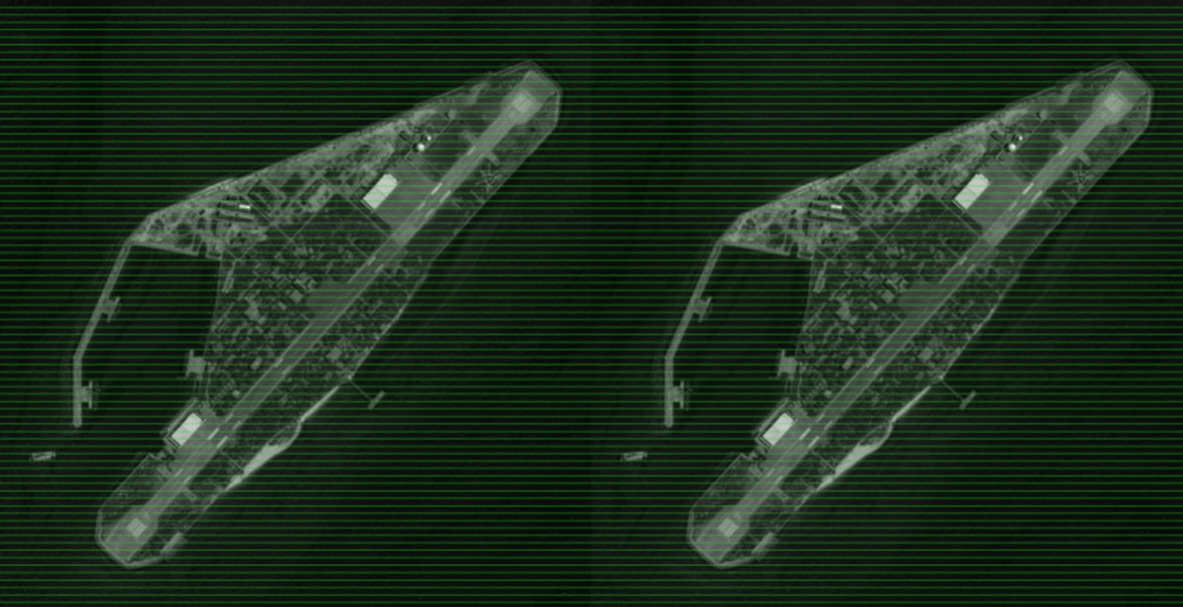
Epipolar image examination diagram. The green line in the figure serves to verify whether the points with the same name in the left and right images lie on the same epipolar line.

The experiments above demonstrate the efficiency and performance of the algorithm proposed in this paper using the Middlebury dataset. This dataset is a standard test set in the field of computer vision and is of great significance for evaluating algorithm performance. However, it mainly consists of close-up and indoor scene images, which differ from remote sensing images. This section analyses the application of the algorithm in remote sensing images. The practical value of the algorithm for 3D reconstruction of remote sensing images is verified through the results of disparity map visualization. This study utilizes remote sensing image data, specifically satellite stereo image pairs obtained from the WorldView-3 satellite in June 2020. These pairs consist of left and right panchromatic images, each boasting a resolution of 19,256 × 14,576. To facilitate this research, the images were resized to a resolution of 3981 × 4082. Figure 9 illustrates the outcome of constructing an epipolar image for analysis, whose parallax up and down can reach 0.1 pixel.

**Figure 10:**
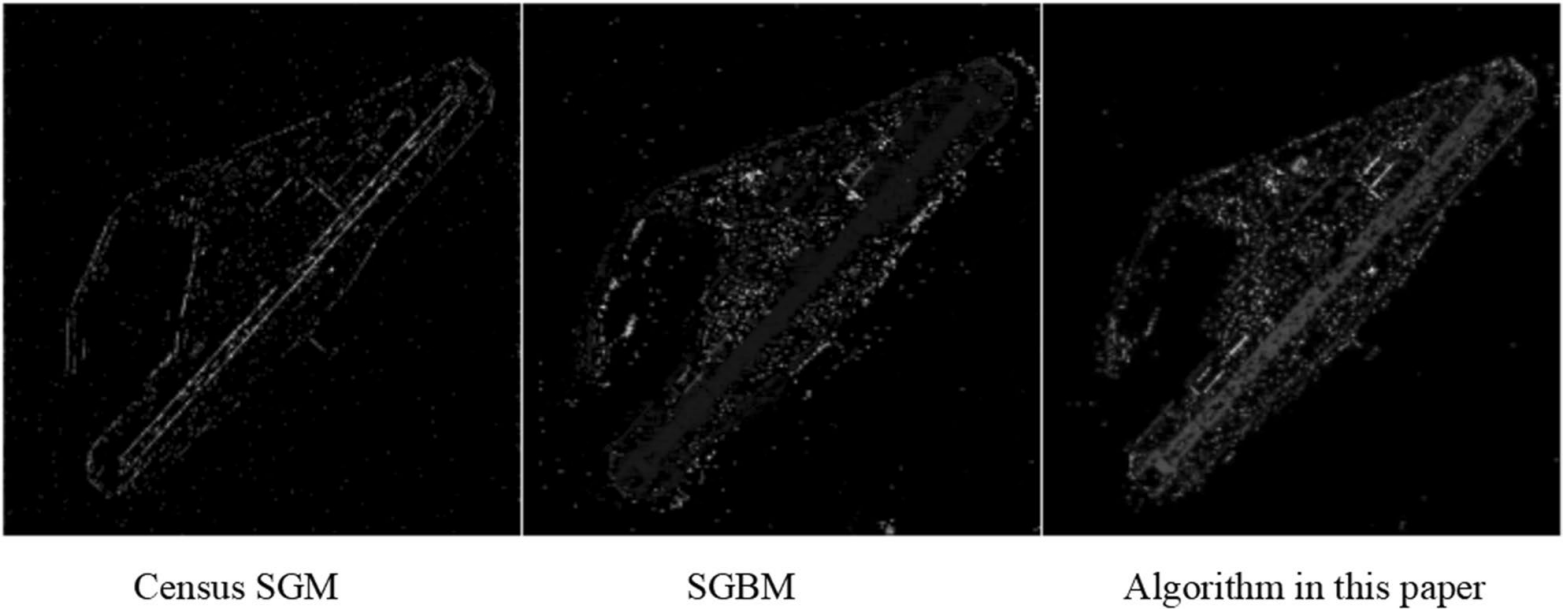
Disparity map of satellite stereo image pairs.

After constructing the epipolar image, three algorithms were employed for semi-global dense matching. Figure 10 displays the resulting disparity map visualisations produced by the three algorithms. The experimental results indicate that the Census SGM algorithm has the lowest mismatch rate on the Middlebury dataset, but it is less effective on remote sensing images due to a large empty area. The SGBM algorithm reduces empty areas, but the consistency of the disparity map with ground object features still requires improvement. In comparison to the previous two algorithms, the proposed algorithm generates a disparity map with fewer empty areas and greater consistency with ground object features. This better reflects object depth information in the image, resulting in improved 3D reconstruction of remote sensing images.

## Conclusion

In this study, the dense matching algorithm for remote sensing images was examined, with a focus on the basic principles of the SGM algorithm, encompassing four key stages: initial cost calculation, cost aggregation, disparity calculation, and disparity optimization. Building on the SGM algorithm, a novel method for dense matching of remote sensing images was developed, integrating CPS denoising. This method assesses the quality of stereo image pairs to determine the necessity of image denoising using the CPS algorithm. Enhancements to the algorithm include image cropping, pixel coordinate transformation, and the construction of an epipolar image with minimized upper and lower disparity. The experimental design involved Python programming to conduct comparative analyses of three distinct algorithms using the test data. The results indicated that the proposed algorithm offers improvements in matching accuracy and efficiency. Moreover, it demonstrates superior performance in image denoising, making it particularly effective for dense matching of remote sensing images. This algorithm holds practical significance for the three-dimensional reconstruction of remote sensing images and enhancing the quality of three-dimensional remote sensing image matching.

## Data Availability

Middlebury 2021 stereo datasets used in the current study can be downloaded from https://vision.middlebury.edu/stereo/data/scenes2021/ and datasets generated during this study are available from the corresponding author on request.
